# Identification of risk factors for pancreatic pseudocysts formation, intervention and recurrence: a 15-year retrospective analysis in a tertiary hospital in China

**DOI:** 10.1186/s12876-018-0874-z

**Published:** 2018-10-01

**Authors:** Jie-hui Tan, Lei Zhou, Rong-chang Cao, Guo-wei Zhang

**Affiliations:** Department of Hepatobiliary Surgery, Nanfang Hospital, Southern Medical University, No.1838, North Guangzhou Avenue, Guangzhou, 510515 People’s Republic of China

**Keywords:** Pancreatic pseudocyst, Alcoholic, Pancreatitis, Outcome, Intervention

## Abstract

**Background:**

Pancreatic pseudocyst (PPC) is a common complication of acute and chronic pancreatitis. To our knowledge no study has systematically reported the risk factors for the formation, intervention and recurrence of PPC. Therefore, the present study aimed to investigate the potential risk factors for PPC, with regards to its formation, intervention and recurrence.

**Methods:**

A database containing 5106 pancreatitis patients was retrospectively analyzed. As a result, a total of 4379 eligible patients were identified and divided into 2 groups: PPC group (group A, *n* = 759) and non-PPC group (group B, *n* = 3620). The PPC group was subdivided into 2 groups: intervention PPC (group C, *n* = 347) and resolution PPC (group D, *n* = 412). The differences in surgical complication and recurrence rates were compared among 347 PPC patients receiving different interventions, including surgical, endoscopic and percutaneous drainages. Furthermore, group C was subdivided into 2 groups: recurrent PPC (group E, *n* = 34) and non-recurrent PPC (group F, *n* = 313). All possible risk factors for PPC formation, intervention and recurrence were determined by multivariate regression analysis.

**Results:**

In this study, PPC was developed in 17.3% (759/4379) of pancreatitis patients. The significant risk factors for PPC formation included alcoholic pancreatitis (OR, 6.332; 95% CI, 2.164–11.628; *p* = 0.031), chronic pancreatitis (CP) (OR, 5.822; 95% CI, 1.921–10.723; *p* = 0.006) and infected pancreatic necrosis (OR, 4.253; 95% CI, 3.574–7.339; *p* = 0.021). Meanwhile, the significant risk factors of PPC patients who received intervention were alcoholic pancreatitis (OR, 7.634; 95% CI, 2.125–13.558; *p* = 0.016), size over 6 cm (OR, 8.834; 95% CI, 2.017–16.649; *p* = 0.002) and CP (OR, 4.782; 95% CI, 1.897–10.173; *p* = 0.038). In addition, the recurrence rate in PPC patients treated with percutaneous drainage was found to be the highest (16.3%) among the three intervention groups. Furthermore, percutaneous drainage was the only risk factor of PPC recurrence (OR, 7.812; 95% CI, 3.109–23.072; *p* = 0.013) identified from this retrospective cohort study.

**Conclusions:**

Alcoholic pancreatitis and CP are the main risk factors for PPC formation and intervention, but not PPC recurrence. A higher recurrence rate is found in PPC patients treated with percutaneous drainage, as compared to endoscopic and surgical interventions.

## Background

According to the revised Atlanta classification [1], acute fluid collections and pseudocyst formation are the most common complications in patients with acute and chronic pancreatitis. A cute peripancreatic fluid collections often lack a wall of granulation or fibrous tissue, which occurred in 30% to 50% of acute pancreatitis (AP) patients within 48 h of AP onset. More than 50% of AP cases disappear spontaneously, or develop into PPC surrounded by a well-defined wall [2]. PPC incidence ranged from 5 to 16% in AP patients, while 20–40% in patients with CP [3–6].

Large PPC is uaually known to cause compressive symptoms and a variety of treatment methods has been proposed such as conservative treatment (watchful monitoring), surgical drainage (open or laparoscopic), percutaneous drainage and endoscopic drainage. Traditionally, the indications for therapeutic intervention of PPC are more than 6 cm in size and persisted for more than 6 weeks. In practice, large pseudocysts are less likely to resolve spontaneously. However, prolonged observation of spontaneous PPC resolution may expose patients to unwarranted risks, including bleeding, perforation, jaundice and infection. Therefore, in order to design effective treatment strategies for patients with PPC, clinical studies should be performed on the basis of an appropriate plan of investigation reflecting the latest scientific and technical knowledge.

To our knowledge, after implementation of the 2012 revised Atlanta classification for AP, the number of retrospective studies focusing on PPC is relatively limited, and most of them has become obsolete. Given these circumstances, further studies are warranted to systematically sought out the incidence, risk factors and intervention effect for PPC. Accordingly, this study aimed to identify the potential risk factors for PPC, with regards to its formation, intervention and recurrence.

## Methods

### Patient identification and selection

A total of 5106 pancreatitis patients (4213 AP cases, 526 CP cases and 367 traumatic pancreatitis cases) hospitalized at NanFang Hospital, Southern Medical University from November 2003 to February 2018 were retrospectively analyzed. All patients were diagnosed and treated according to the guidelines of the Pancreatic Surgical Science Section of the Chinese Medical Association Surgery Branch in 2014, and were graded according to the 2012 revised Atlanta classification for AP. According to the 2012 revised Atlanta classification for AP, severity is classified as mild, moderate or severe. Mild acute pancreatitis has no organ failure, local or systemic complications. Moderately severe acute pancreatitis is defined by the presence of transient organ failure, local complications or exacerbation of co-morbid disease. Severe acute pancreatitis is defined by persistent organ failure, that is, organ failure > 48 h [7]. All interventions were performed by or under the supervision of consultant surgeons and their assistants. PPC resection and cyst-enteric bypass were the primary treatment methods in these patients. The study protocol was approved by the ethics committee of the same hospital.

**Fig. 1:**
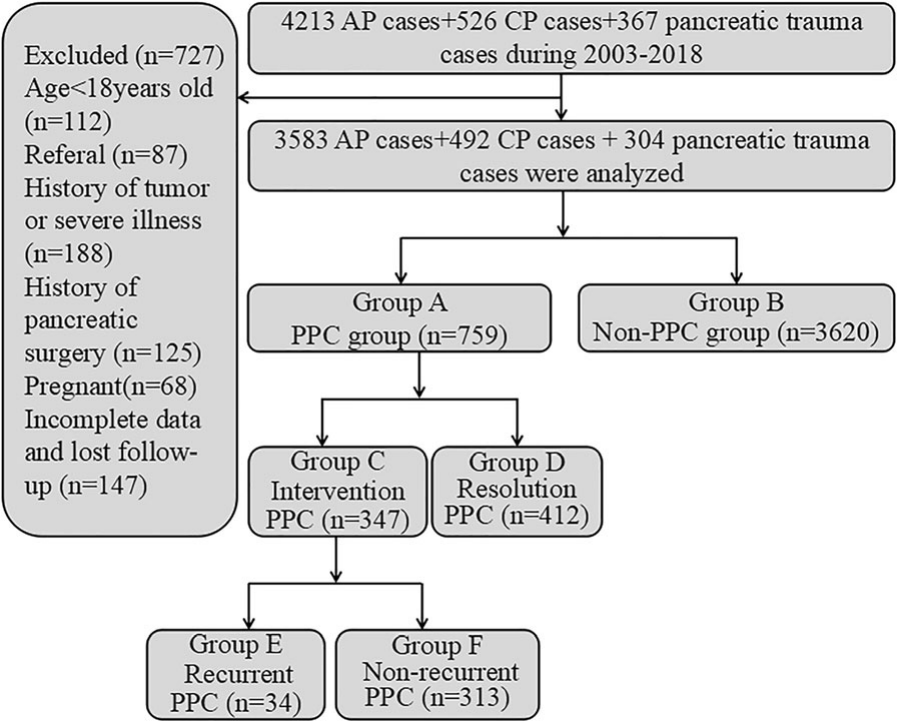
A flow chart showing patients included in this study, proportion of patients in each group and reasons for exclusion

Among these patients, 4379 pancreatitis cases fulfilled the in-teamed standard and were divided into PPC group (group A, *n* = 759) and non-PPC group (group B, *n* = 3620). PPC was defined according to the revised Atlanta criteria. Group A was further divided into 2 groups: intervention PPC (group C, *n* = 347) and resolution PPC (group D, *n* = 412). Similarly, group C was divided into 2 groups: recurrent PPC (group E, *n* = 34) and non-recurrent PPC (group F, *n* = 313) (Fig. 1). All PPC patients were followed up for at least 6 weeks after diagnosis, while all intervention patients were followed up for at least 3 months after treatment.

### Statistical analysis

All statistical analyses were performed with SPSS software (SPSS version 22.0, Chicago, IL, USA). Inter-group comparisons were determined by Pearson’s chi-square test, Student t test or Mann-Whitney U test, whenever appropriate. Univariate and multivariate logistic regression analyses were used to investigate the risk factors for the formation, intervention and recurrence of PPC. *P* values of less than 0.05 were considered statistically significant.

## Results

The clinical characteristics of the 4379 pancreatitis patients are summarized in Table 1. PPC was developed in 17.3% (759/4379) of pancreatitis patients. Intriguingly, alcoholic etiology (OR, 6.332; 95% CI, 2.164–11.628; *p* = 0.031), CP (OR, 5.822; 95% CI, 1.921–10.723; *p* = 0.006) and infected pancreatic necrosis (IPN) (OR, 4.253; 95% CI, 3.574–7.339; *p* = 0.021) were revealed as significant risk factors for PPC formation (Table 1). Through multivariate analysis, the independent risk factors for PPC patients who required intervention were found to be alcoholic etiology (OR, 7.634; 95% CI, 2.125–13.558; *p* = 0.016), CP (OR, 4.782; 95% CI, 1.897–10.173; *p* = 0.038) and size over 6 cm (OR, 8.834; 95% CI, 2.017–16.649; *p* = 0.002) (Table 2). Additionally, there were statistically significant differences in the recurrence rates among endoscopic, surgical and percutaneous drainage groups, but not complications. The recurrence rate of PPC treated with percutaneous drainage was 16.3%, which ranked the highest among the three intervention groups (Table 3). Notably, percutaneous drainage (OR, 7.812; 95% CI, 3.109–23.072; *p* = 0.013) was the only independent risk factor for PPC recurrence, as assessed by multivariate analysis (Table 4).

**Table 1:** Univariate and multivariate regression analyses of risk factors associated with PPC formation

Variable	Univiarite analysis				Multivariate analysis	
	Total (*n* = 4379)	Group A (*n* = 759)	Group B (*n* = 3620)	*P*-value	OR (95% CI)	*P*-value
Age (years)	47.82 ± 15.31	47.33 ± 14.65	48.12 ± 15.74	0.523		
Sex (male/female)	2788/1591	435/324	2253/1467	0.096		
BMI	24.71 ± 4.85	24.28 ± 4.76	23.87 ± 5.13	0.329		
Smoking (yes/no)	1343/3036	252/507	1091/2529	0.096		
Comorbidity						
Hypertension	425	78	347	0.559		
Diabetes	505	83	412	0.724		
Respiratory diseases	214	35	179	0.698		
Liver diseases	147	34	113	0.061		
Pancreatitis (acute/chronic)	3887/492	654/105	3233/387	0.013^a^	5.822 (1.921-10.723)	0.006^a^
Recurrent pancreatitis (yes/no)	861/3518	143/616	718/2917	0.565		
Symptoms						
Pain	4334	748	3586	0.028^a^	1.557 (0.891-3.425)	0.067
Fever	751	112	639	0.054		
Etiology						
Biliary	1923	354	1569	0.096		
Alcoholic	785	163	622	0.005^a^	6.332 (2.164-11.628)	0.031^a^
Trauma	304	42	262	0.093		
Hyperglycemia	342	49	293	0.126		
Post ERCP	577	84	493	0.059		
Idiopathic	448	67	381	0.161		
Lab examination						
Amylase (U/L)	859.37 ± 612.35	831.22 ± 579.28	864.19 ± 634.56	0.574		
WBC (10^9^/L)	13.79 ± 7.36	14.58 ± 8.24	12.46 ± 7.10	0.218		
CRP (mg/L)	82.63 ± 28.52	86.48 ± 32.67	78.44 ± 26.36	0.227		
TBIL (umol/L)	52.21 ± 33.62	54.37 ± 36.27	51.46 ± 31.70	0.232		
IPN (%)	337 (7.7)	78 (10.3)	259 (7.2)	0.003^a^	4.253 (3.574-7.339)	0.021^a^
Antibiotics (Yes/No)	3782/592	644/115	3138/477	0.152		
Somatostatin (Yes/No)	4201/178	725/34	3476/144	0.525		

Data are expressed as n (%) or mean ± standard

^a^Statistically significant results (*P* < 0.050)

**Table 2:** Univariate and multivariate regression analyses of risk factors associated with PPC which needs intervention

Variable	Univiarite analysis			Multivariate analysis	
	Group C (*n =* 347)	Group D (*n* = 412)	*P*-value	OR (95% CI)	*P*-value
Age (years)	46.85 ± 15.19	48.04 ± 14.27	0.264		
Sex (male/female)	193/154	242/170	0.387		
BMI	24.59 ± 4.62	24.13 ± 4.83	0.614		
Smoking (yes/no)	115/232	137/275	0.974		
Comorbidity					
Hypertension	32/315	46/366	0.380		
Diabetes	39/308	44/368	0.806		
Respiratory diseases	13/336	22/390	0.289		
Liver diseases	14/333	20/392	0.586		
Pancreatitis (acute/chronic)	285/62	369/43	0.003^a^	4.782 (1.897-10.173)	0.038^a^
Recurrent pancreatitis (yes/no)	68/279	75/337	0.625		
Symptoms					
Pain	341	407	0.554		
Fever	48	64	0.510		
Etiology					
Biliary	149	205	0.061		
Alcoholic	89	74	0.010^a^	7.634 (2.125-13.558)	0.016^a^
Trauma	18	24	0.701		
Hyperglycemia	23	26	0.859		
Post ERCP	37	47	0.745		
Idiopathic	31	36	0.905		
Lab examination					
Amylase (U/L)	912.47 ± 674.63	819.23 ± 626.37	0.172		
WBC (10^9^/L)	14.71 ± 8.65	13.85 ± 8.23	0.384		
CRP (mg/L)	82.05 ± 28.39	88.72 ± 31.33	0.271		
TBIL (umol/L)	56.42 ± 34.71	52.93 ± 38.37	0.325		
Time from pancreatitis to pseudocyst (weeks)	8.47 ± 1.78	9.12 ± 2.05	0.311		
Location			0.043^a^	2.534 (0.892-3.665)	0.083
Head	129	183			
Body/Tail	218	229			
Number			0.037^a^	2.754 (0.821-4.378)	0.064
Single	183	186			
Multiple	164	226			
Size			0.011^a^	8.834 (2.017-16.649)	0.002^a^
≥ 6 cm	144	134			
< 6 cm	203	278			
IPN	44	34	0.045^a^	1.811 (0.893-3.552)	0.056
Antibiotics (Yes/No)	302/45	342/70	0.124		
Somatostatin (Yes/No)	331/16	394/18	0.872		

Data are expressed as n (%) or mean ± standard

^a^Statistically significant results (*P* < 0.050)

**Table 3:** Comparison of complications of 347 PPC intervention patients according to different intervention methods

Total (*n =* 347)	Endoscopic	Surgical	Percutaneous drainage	*P*-value
	48	164	135	
Infection	5	13	22	0.076
Hemorrhage	2	5	5	0.914
Anastomotic/Percutaneous Leakage	3	4	7	0.342
Pancreatitis exacerbation	2	2	1	0.219
Organ failure	1	2	2	0.906
Mortality	1	1	3	0.464
Recurrence	4	8	22	0.004^a^

^a^Statistically significant results (*P <* 0.050)

**Table 4:** Univariate and multivariate regression analyses of risk factors associated with PPC recurrence

Variable	Univiarite analysis			Multivariate analysis	
	Group E (*n =* 34)	Group F (*n =* 313)	*P*-value	OR (95% CI)	*P*-value
Age(years)	47.33 ± 15.42	46.42 ± 14.82	0.317		
Sex (male/female)	19/15	174/139	0.974		
BMI	24.12 ± 4.34	24.69 ± 4.82	0.538		
Smoking (yes/no)	8/26	107/206	0.210		
Comorbidity					
Hypertension	4	28	0.589		
Diabetes	5	34	0.500		
Respiratory diseases	1	12	0.795		
Liver diseases	0	14	0.208		
Pancreatitis (acute/chronic)	27/7	258/55	0.663		
Recurrent pancreatitis (yes/no)	11/23	57/256	0.048^a^	2.017 (0.926-4.173)	0.063
Symptoms					
Pain	33	308	0.568		
Fever	8	40	0.085		
Etiology					
Biliary	13	136	0.560		
Alcoholic	7	82	0.477		
Trauma	3	15	0.314		
Hyperglycemia	2	21	0.854		
Post ERCP	3	34	0.714		
Idiopathic	6	25	0.061		
Lab examination					
Amylase (U/L)	958.26 ± 662.37	872.51 ± 652.46	0.142		
WBC (10^9^/L)	13.67 ± 8.24	15.21 ± 8.32	0.254		
CRP (mg/L)	79.32 ± 27.61	83.23 ± 29.39	0.371		
TBIL (umol/L)	57.72 ± 33.69	56.10 ± 35.32	0.652		
Time from pancreatitis to pseudocyst	8.74 ± 2.16	8.37 ± 1.85	0.725		
Location			0.099		
Head	14	115			
Body/Tail	20	198			
Number			0.325		
Single	15	168			
Multiple	19	145			
IPN	8	36	0.045^a^	1.483 (0.875-3.262)	0.083
Antibiotics (Yes/No)	31/3	271/42	0.449		
Somatostatin (Yes/No)	33/1	298/15	0.625		
Intervention methods			0.004^a^	7.812 (3.109-23.072)	0.013^a^
Endoscopic drainage (%)	4 (8.3%)	44 (91.7%)			
Surgical drainage (%)	8 (4.9%)	156 (95.1%)			
Percutaneous drainage (%)	22 (16.4%)	113 (83.6%)			

Data are expressed as n (%) or mean ± standard

^a^Statistically significant results (*P <* 0.050)

## Discussion

PPC, a begin complication of pancreatitis, can be predictors of a malignant outcome, especially among patients with severe AP. The two main indications for some type of invasive drainage procedure are persistent patient symptoms or the presence of complications such as bleeding, infection, gastric outlet and biliary obstruction [8]. To date, the guidelines on minimally invasive management of PPC demonstrated a lack of consensus in clinical recommendations, and few recommendations have been graded according to the strength of supporting evidence. The identification and prediction of risk factors for PPC formation, intervention and recurrence may help to distinguish the high-risk PPC group from patients with pancreatitis. Thus, early detection and treatment can be considered for patients at high-risk of PPC. Additionally, identification of risk factors may reduce surgical adverse events, avoid delay in inappropriate interventions and improve the prognosis of PPC patients.

In the present study, data of 5106 pancreatitis patients was retrieved from a prospective database and was retrospectively analyzed. After reviewing the English-language articles published in PubMed with MeSH terms of “pancreatitis”, “pancreatic pseudocyst”, “pancreatic necrosis”, “infected pancreatic necrosis”, or “pancreatic fluid collections”, we believed that this study contained the largest population of PPC patients at a single center, reporting the risk factors of PPC formation, intervention and recurrence. Alcoholic and chronic pancreatitis remained the main risk factors for PPC formation and intervention. Although the recurrence rate of PPC treated with percutaneous drainage was ranked the highest, there was no difference in the rate of complications among the three types of interventions.

Biliary pancreatitis is ranked the most common cause of PPC among Asian countries, followed by alcoholic pancreatitis. However, more severe forms of AP and local complication, such as pseudocyst formation, have been associated with alcoholic AP compared to biliary AP [9]. Alcohol acts to worsen pancreatitis by its effects on pancreatic mitochondria to promote necrosis, which has been proved by in vitro experiments and clinical research [10, 11]. Besides, nonalcoholic acute pancreatitis is associated with a lower incidence of pseudocyst formation when compared with acute alcoholic pancreatitis. Alcoholism etiology has been reported as one of the risk factors for pancreatic fluid collections [12]. On the other hand, a high incidence of pseudocyst formation has been found among patients with CP. A multicenter study from China reported that 26.25% of CP patients are more likely to develop pseudocysts [13]. PPC due to CP, is often accompanied by secondary complications, including duodenal and/or biliary obstruction, splenic vein thrombosis and rarely infection [14]. These complications are primarily treated by surgery and less amenable to endoscopic therapy, especially for common bile duct stricture, main pancreatic duct obstruction and pseudocysts [15]. Furthermore, alcoholism exhibits a worse effect on pancreatic function and is the most common cause of CP. These findings suggest the importance of alcoholic pancreatitis and CP as new combinational risk factor for PPC formation.

IPN, a local complication of severe AP, is commonly accompanied with PPC, due to the collection of pancreatic necrotic tissues by PPC. Typically, pancreatic necrosis is a late complication of AP, resulting in considerable morbidity and mortality. The necrotic pancreatic tissues can remain solid or liquefy, and remain sterile or become infected. Among the patients with necrotizing pancreatitis, 33% of them may develop infected necrosis. The prevalence of organ failure in necrotizing pancreatitis is 54% and even higher among patients with infected necrosis [16]. To the best of our knowledge, no studies have reported on the association between IPN and PPC. The present study revealed that IPN was significantly correlated with PPC formation (OR, 4.253; 95% CI, 3.574–7.339; *p* = 0.021). Therefore, it is noted that an active and effective treatment for IPN can prevent the development of PPC, improve the prognosis of pancreatitis patients, and even lower the morbidity and mortality rate.

The surgical techniques and timing of treatment for PPC are still in debate. Most previous studies have shown that PPC larger than 5 or 6 cm are less likely to resolve spontaneously. The intervention for patients with a small pseudocyst and mild symptoms can be delayed for a further 3 months, since the spontaneous resolution of PPC may still occur [17]. A prolonged period of “wait-and-see” policy for more than 6 weeks is suggested for patients with asymptomatic pseudocyst, especially for a single lesion [6]. Spontaneous resolution has occurred in 40% to 50% of PPC patients with no major complications during the period of active observation. As a consequence, intervention is warranted if the patient is symptomatic, a progressive increase in PPC size or if complications occur [18]. However, it has been reported that a delay of surgical intervention in PPC may contribute to higher incidences of postoperative complications, readmission, morbidity, and mortality. Moreover, the increasing application of nonsurgical interventions may require a further evaluation [19]. The concept of practice is that the wait-and-see policy should be carried out for more than 4 to 6 weeks until the appearance of spontaneous remission, unless PPC is associated with other symptoms or complications. Generally, chronic pseudocyst encapsulated with a thicker and more well-defined wall than acute pseudocyst [20]. The surgical intervention is usually performed on PPC with a wall thickness of greater than 1 mm. In addition, patients with first-attack AP and fluid collections at discharge should be examined by ultrasonography at a 3-month follow-up, in order to detect the presence of asymptomatic complications such as PPC.

**Fig. 2:**
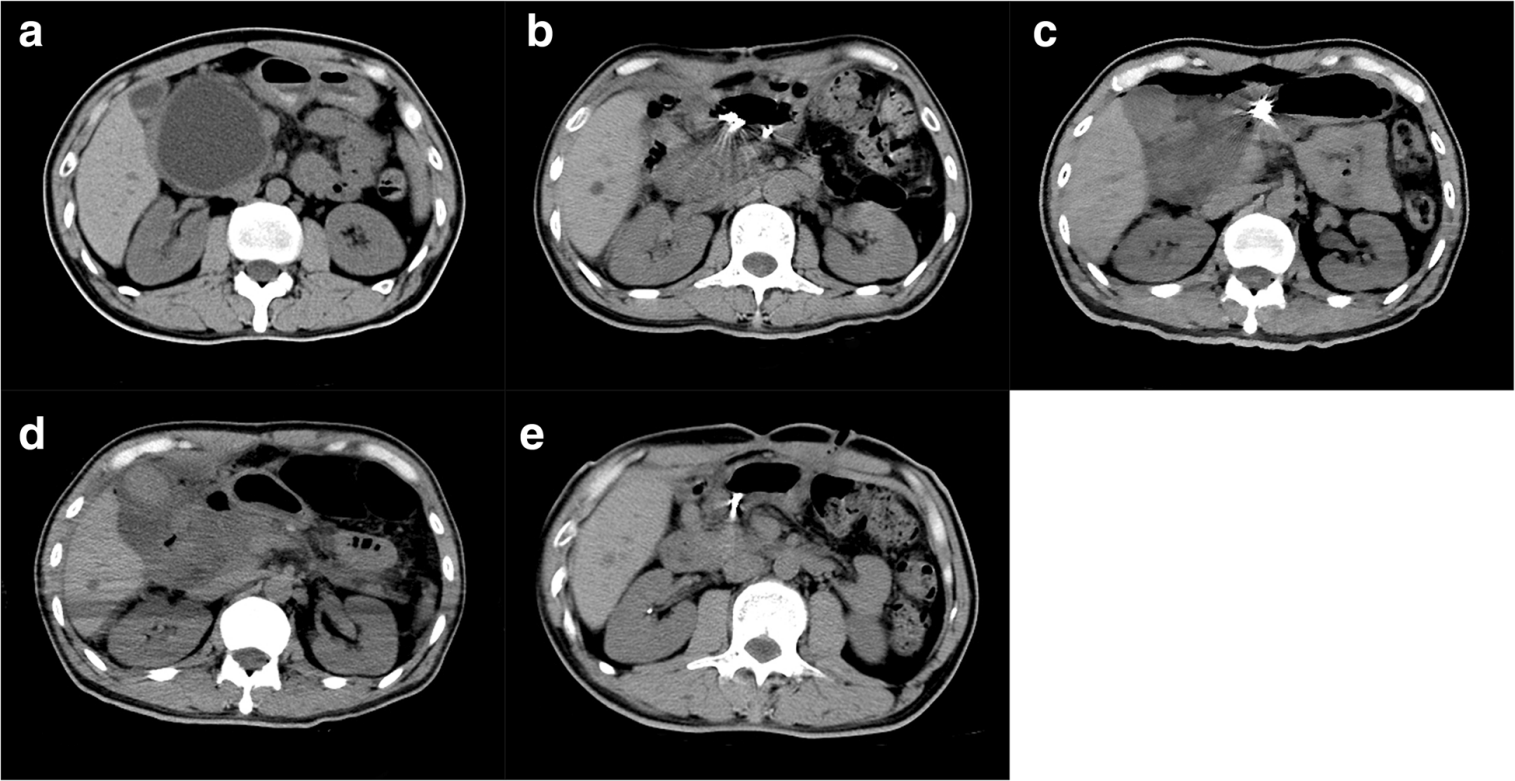
A PPC case who first received endoscopic cystgastrostomy complicated with anastomotic leakage. The patient received surgical drainage 4 days after endoscopy. **a** Abdominal CT scan showing a 8-cm PPC; **b** Pneumoperitoneum occurred on day 1 post-endoscopy; **c** Seroperitoneum occurred on day 2 post-endoscopy; **d** Peritonitis occurred on day 3 post-endoscopy; **e** Pneumoperitoneum and seroperitoneum disappeared in 1 month since surgical drainage

**Fig. 3:**
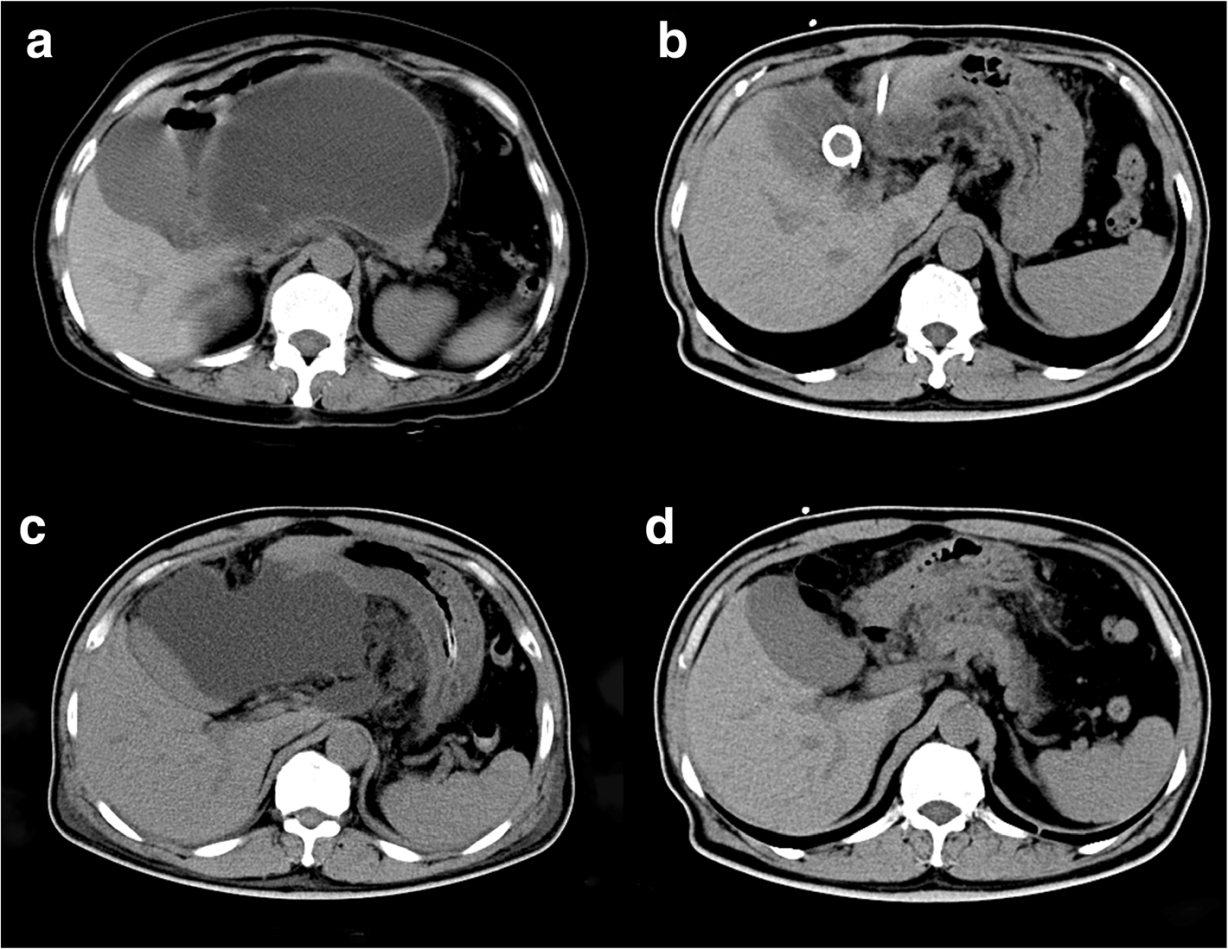
A PPC case who first received percutaneous drainage recurred, then was cured by surgical drainage. **a** Abdominal CT scan showing a 13-cm PPC; **b** PPC resolution on day 7 post-percuataneous drainage; **c** PPC recurrence in 2 months since percutaneous drainage; **d** PPC disappeared in 1 month since surgical drainage

Thus far, there have been no prospective studies comparing the effects of different intervention techniques (i.e. endoscopic drainage, percutaneous drainage and surgical drainage) on the complication and recurrence rates of PPC. The success rate of PPC after endoscopic drainage is considerably variable, most likely due to the presence of heterogeneity among patient populations and intervention types [21]. Surgery is no longer used as a sole treatment for PPC, ever since the emergence of alternative first-line therapy at most centers. Although both endoscopic and surgical drainages have demonstrated comparable success rates, there is a lack of published data regarding the optimal intervention for PPC patients [22]. Some patients may require multiple endoscopic procedures, and the decision to pursue endoscopic therapy depends on patient preference, underlying medical conditions and whether an additional endoscopic procedure is feasible. In addition, percutaneous drainage has been applied in patients with acute pseudocyst or the presence of physiologic exhaustion or comorbid conditions that prevent surgical intervention [23]. Percutaneous drainage provides a convenient alternative to patients, practitioners and physicians. However, several studies reported an equal effectiveness of percutaneous, endoscopic and surgical drainage [22, 24, 25]. In the present study, surgical drainage has the lowest recurrence rate as compared to endoscopic and percutaneous drainages (OR, 7.812; 95% CI, 3.109–23.072; *p* = 0.013). For the complication and recurrence rates of PPC among the three intervention groups, surgery is considered as the last remedial step (Figs. 2 and 3). Despite a higher recurrence rate of PPC in percutaneous drainage group, especially for children, PPC can often be managed without surgery, regardless of its size or complexity [26].

The advancement of new techniques in endoscopic and laparoscopic approaches have reduced the postoperative morbidity and mortality rates of PPC patients. Given that severe complications may occur after the procedure (Fig. 2), endoscopic drainage is recommended to be performed at tertiary-care center, by a surgeon with expertise in pancreatic surgery [27]. Both laparoscopic and open pancreatic cystgastrostomy have high primary success rates than endoscopic internal drainage, although repeated endoscopic cystgastrostomy offers a better success rate for selected PPC patients [28]. There have been various surgical approaches for treating PPC, but none of them are used as gold standards, as the choice of treatment is much dependent on the surgeon‘s experience and the clinical characteristics of patient. For patients with symptomatic CP, a multidisciplinary approach appears to have low threshold to surgical intervention, since long-term pain relief is accomplished more often after surgical treatment than after endoscopic treatment [29]. Surgical treatment for PPC patients consistes of open and laparoscopic approaches and includes the following: open drainage, cystogastrostomy, cystojejunostomy, distal pancreatectomy, PPC resection and pancreato-jejunostomy [30]. The laparoscopic approach to cystogastrostomy for PPC is associated with a shorter operating time, a smoother and more rapid postoperative recovery, and a shorter length of hospital stay compared to open surgery. Hence, the laparoscopic approach should be considered as the preferred treatment modality for PPC, when laparoscopic expertise is available [31].

## Conclusion

Alcoholic and chronic pancreatitis may serve as the major risk factors for PPC formation and intervention. Moreover, percutaneous drainage is the only independent risk factor for PPC recurrence. The main limitations of this study include its retrospective design and single-institution nature. Therefore, future multi-institutional prospective studies are warranted to provide additional evidence supporting the risk factors for PPC, and the research results should be incorporated into clinical practice guidelines.

## Abbreviations

AP: Acute pancreatitis
CP: Chronic pancreatitis
IPN: Infected pancreatic necrosis
PPC: Pancreatic pseudocyst
